# Transcranial magnetic stimulation as a tool to understand genetic conditions associated with epilepsy

**DOI:** 10.1111/epi.16634

**Published:** 2020-08-12

**Authors:** Katri Silvennoinen, Simona Balestrini, John C. Rothwell, Sanjay M. Sisodiya

**Affiliations:** ^1^ Department of Clinical and Experimental Epilepsy UCL Queen Square Institute of Neurology London UK; ^2^ Chalfont Centre for Epilepsy Chalfont St. Peter UK; ^3^ Sobell Department of Motor Neuroscience and Movement Disorders Department of UCL Queen Square Institute of Neurology London UK

**Keywords:** GABA, gene, inhibition, syndrome, variant

## Abstract

Advances in genetics may enable a deeper understanding of disease mechanisms and promote a shift to more personalised medicine in the epilepsies. At present, understanding of consequences of genetic variants mainly relies on preclinical functional work; tools for acquiring similar data from the living human brain are needed. Transcranial magnetic stimulation (TMS), in particular paired‐pulse TMS protocols which depend on the function of cortical GABAergic interneuron networks, has the potential to become such a tool. For this report, we identified and reviewed 23 publications on TMS studies of cortical excitability and inhibition in 15 different genes or conditions relevant to epilepsy. Reduced short‐interval intracortical inhibition (SICI) and reduced cortical silent period (CSP) duration were the most commonly reported findings, suggesting abnormal GABA_A_‐ (SICI) or GABA_B_ergic (CSP) signalling. For several conditions, these findings are plausible based on established evidence of involvement of the GABAergic system; for some others, they may inform future research around such mechanisms. Challenges of TMS include lack of complete understanding of the neural underpinnings of the measures used: hypotheses and analyses should be based on existing clinical and preclinical data. Further pitfalls include gathering sufficient numbers of participants, and the effect of confounding factors, especially medications. TMS‐EEG is a unique perturbational technique to study the intrinsic properties of the cortex with excellent temporal resolution; while it has the potential to provide further information of use in interpreting effects of genetic variants, currently the links between measures and neurophysiology are less established. Despite these challenges, TMS is a tool with potential for elucidating the system‐level in vivo functional consequences of genetic variants in people carrying genetic changes of interest, providing unique insights.

**Key Points**

- To translate epilepsy genetics to precision medicine, tools for interpreting disease mechanisms at the level of the individual patient are needed.
- TMS‐EMG paired‐pulse measures, which reflect the function of GABAergic cortical interneuron circuits, may be particularly relevant for some genetic epilepsies.
- For several reviewed conditions, TMS‐EMG provides evidence of altered inhibition, in keeping with hypotheses of the effects of the conditions on the GABA system.
- More research is required to expand and corroborate these findings, including the role of TMS‐EEG.

## 1 INTRODUCTION

The epilepsies are a heterogeneous group of conditions characterized by predisposition to recurrent seizures^1^ and involve alterations in excitation‐inhibition balance of cerebral networks.^2^ In over a third of patients, seizures persist despite appropriate medical treatment.^3^ In this setting, a precision medicine approach, where a patient would be offered a treatment most likely to be effective in their particular condition, would be especially valuable.^4^

The rapidly increasing knowledge of the genetics of epilepsies has contributed to the understanding of their pathophysiological mechanisms and improved diagnostic ability on the individual level.^5^ To facilitate the translation of genetics to precision medicine, further study of the functional consequences of genetic variants and better understanding of interindividual variation in phenotypes are required.^4^ Whilst traditional preclinical models are powerful tools to reveal basic underlying mechanisms, they have inherent limitations in the context of in vivo whole‐organism processes in humans, and there is a pressing need for tools for interpreting disease mechanisms at the level of the individual patient.^6^

**FIGURE 1:**
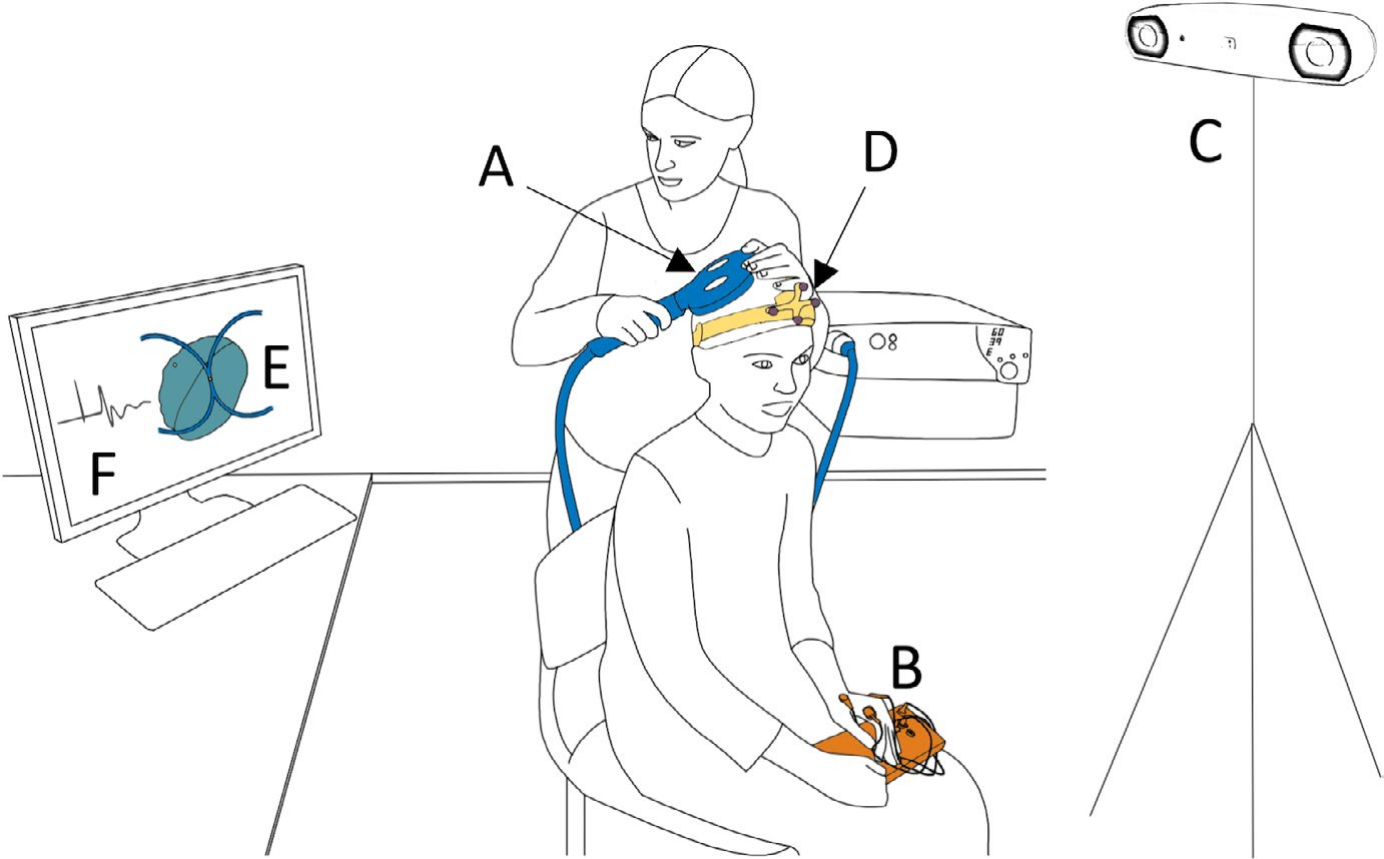
Setup for TMS‐EMG. The magnetic pulse is delivered through the figure‐of‐eight coil (A), which is held over the primary motor cortex. EMG is recorded from contralateral intrinsic hand muscles using surface electrodes (B). A neuronavigation system consisting of an infrared camera (C) and coil and subject trackers (D) is used to visualise the position of the coil with respect to the brain (E). The evoked EMG trace (F) is also displayed on a computer screen

**FIGURE 2:**
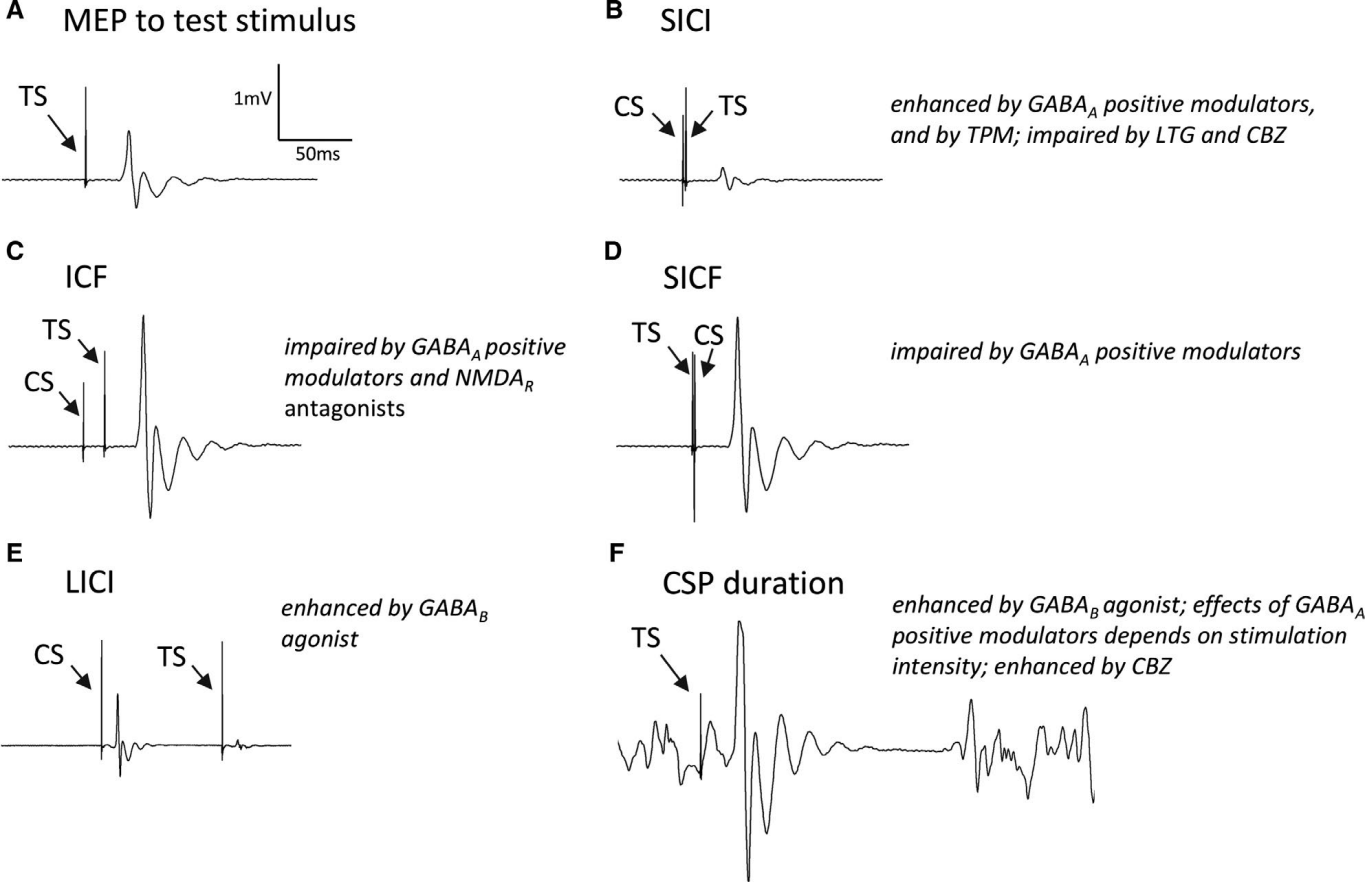
TMS‐EMG paradigms, and effects of GABA modulators and common AEDs. A: A motor evoked potential (MEP) evoked by a test stimulus alone. B: Short‐interval intracortical inhibition (SICI) is GABA_A_ dependent and is elicited when a conditioning stimulus (CS) is delivered 1‐5 ms before the test stimulus (TS). C: When the inter‐stimulus interval is longer, 10‐15 ms, there is facilitation instead (intracortical facilitation; ICF), D: Short‐interval intracortical facilitation (SICF) occurs when a conditioning stimulus follows the test stimulus at specific intervals associated with I‐wave periodicity. E: Long‐interval intracortical inhibition (LICI) is seen when a conditioning stimulus precedes the test stimulus by 60‐200 ms; this is GABA_B_‐dependent. F: Cortical silent period (CSP) presents the interruption of voluntary muscle contraction following the test stimulus; this is GABA_B_‐dependent. Abbreviations: CBZ, carbamazepine; LTG, lamotrigine; TPM, topiramate

Transcranial magnetic stimulation (TMS) is a non‐invasive means of studying cortical excitability employing electromagnetic induction (Figure 1).^7^ Paired‐pulse measures (Figure 2), which reflect the function of GABA‐dependent cortical interneuron circuits,^7^, ^8^ could be especially relevant in the context of some genetic epilepsies, in which abnormal interneuron function in implicated.^9^ A number of studies have attempted to identify TMS‐based biomarkers for diagnosis or prediction of treatment response in the epilepsies.^10^, ^11^ In general, these studies have involved relatively heterogenous patient groups, which may have contributed to the paucity of clinically adopted markers.^10^, ^11^

Studying TMS in genetically‐defined patient groups may decrease inter‐individual variability and increase power for detecting clinically useful signatures of pathophysiological processes yielding biomarkers for diagnosis and treatment of these conditions. This approach has already been employed for several genetic conditions of relevance to epilepsy, which we now review.

## 2 BASIC TMS MECHANISMS AND PARAMETERS

Transcranial magnetic stimulation involves using a time‐varying magnetic field generated over the head to induce an electric field reaching the cerebral cortex (Figure 1). When sufficiently strong, the electric field depolarises cortical neurons. TMS targets neural populations depending on their axonal orientation with respect to the direction of the induced current, and on stimulation intensity. The neural signal is propagated transsynaptically to anatomically connected areas.

A number of factors affect cortical excitability as measured by TMS. In the motor system, which has been most extensively studied so far, GABAergic inhibitory interneurons are thought to be involved in modulation of I‐waves, which propagate down the corticospinal tract following stimulation of the motor cortex.^12^ Thus the function of GABAergic neurons themselves has an impact on responses to TMS, and in some conditions, GABAergic neuronal function is compromised (see below).

When coupled with electromyography (EMG), the TMS pulse is applied over the primary motor cortex, typically the hand area (Figure 1). As a basic measure of cortical excitability, the resting motor threshold (rMT) is defined as the minimum intensity required to produce motor evoked potentials (MEPs) in at least five out of ten trials.^7^ Stimulus intensities in other paradigms are often determined with respect to the rMT (sub‐ or suprathreshold).

In paired‐pulse TMS (Figure 2), a conditioning stimulus (CS) is used to modulate the MEP evoked by the subsequent test stimulus (TS).^7^ The effect of the CS on the MEP evoked by the test stimulus is influenced by the inter‐stimulus interval (ISI).^7^ Other factors may also have an influence. The effects are generally expressed as the ratio between conditioned and unconditioned MEP amplitudes.^7^ Short‐interval intracortical inhibition (SICI) is elicited with a subthreshold CS and ISIs of 1‐5 ms^13^; it is considered to reflect GABA_A_ergic inhibition.^8^ Intracortical facilitation (ICF; ISI is 10‐15 ms) is thought to reflect a net effect of increased facilitation over GABA_A_‐mediated inhibition.^8^, ^13^ In contrast, short‐interval intracortical facilitation (SICF) is elicited when a near‐threshold CS follows the TS at intervals of around 1.5 ms, corresponding to those observed between I‐waves.^14^ Long‐interval intracortical inhibition (LICI) is elicited when a suprathreshold CS precedes the TS by 60‐200 ms and is associated with GABA_B_ergic inhibition.^8^, ^15^ The duration of the cortical silent period (CSP), elicited when a single suprathreshold single pulse is targeted at the cortical hotspot of a contracted muscle, may also be used as a measure of GABA_B_ergic inhibition.^7^ These paradigms are discussed in more detail in the Material S1.

**FIGURE 3:**
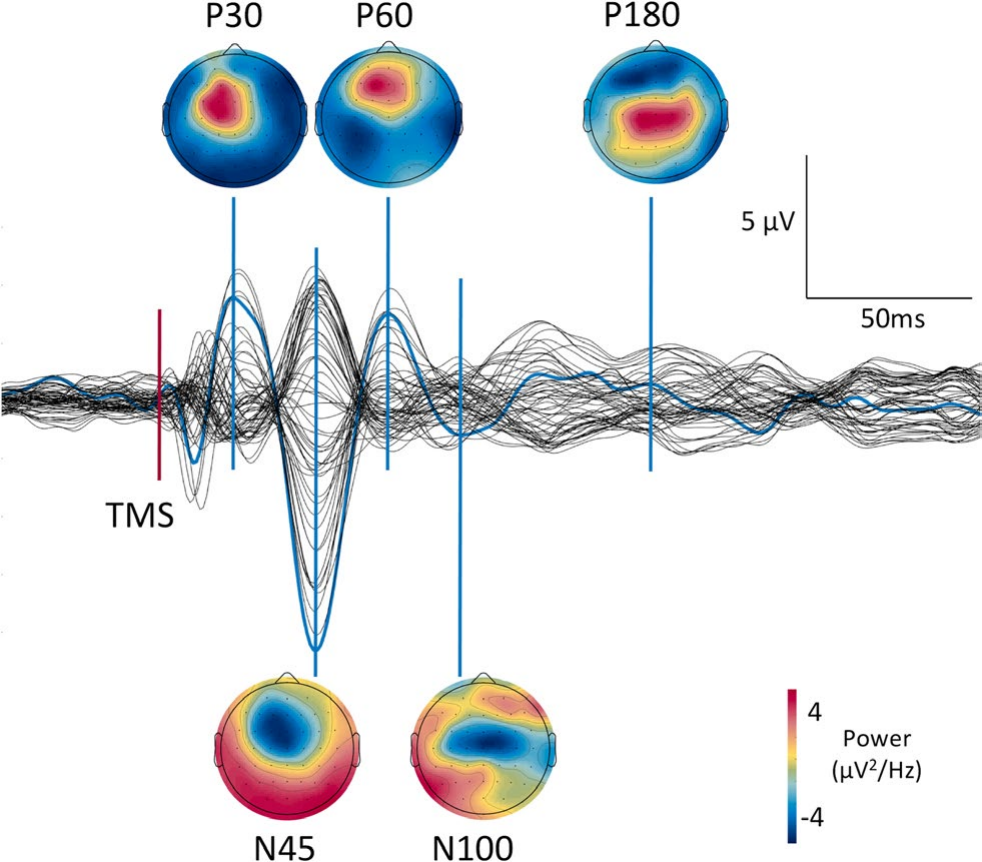
Butterfly plot of a TMS‐evoked potential (TEP) from stimulation of left premotor cortex. Channels are referenced to average. Channel FCz is highlighted in blue. Components are designated by their polarity and latency. Compared to earlier components, N100 and P180 are less well defined. The scalp maps show the potential distribution and power for the latencies associated with these components. Abbreviations: μV, microvolt; Hz, hertz

EEG may be used to measure oscillatory activity of the cortex, which reflects excitatory and inhibitory postsynaptic potentials generated by populations of cortical neurons.^16^ A TMS pulse leads to non‐physiological perturbation (simultaneous depolarisation) affecting a large population of cortical neurons, resetting their endogenous oscillatory activity; EEG may be used to study the evoked oscillatory response (Figure 3).^16^ The frequency and waveform of this TMS‐EEG response are reproducible and characteristic of the stimulated area.^17^ However, it is important to note that particularly the later components of the TEP (>100 ms after the TMS pulse) may be contaminated by brain activity resulting from the audible noise and scalp sensation that TMS produces, although these can be reduced with appropriate adjustments to technique.^18^, ^19^ Studying the propagation of the TEP may provide an additional measure of cortical function, specifically the effective connectivity within ﻿cortico‐cortical and cortico‐subcortical networks.^16^ See Material S1 for more details.

### 2.1 The effect of antiepileptic drugs on TMS parameters

A summary of the effects of major classes of antiepileptic drugs in healthy controls is presented in Table S1. The effect of introduction of lamotrigine, a sodium channel blocker, on increasing rMT was also demonstrated in people with epilepsy whereas no effect was seen on CSP.^20^

## 3 LITERATURE REVIEW

A literature search was conducted using PubMed for “transcranial magnetic stimulation” AND (“genetics” OR “genes” OR “syndrome” OR “gene”) NOT rTMS. See Material S1 for more details. The experimental details and findings are summarised in Table 1. The conditions are grouped below by presumed main mechanisms implicated, where possible.

**TABLE 1:** Summary of TMS‐EMG findings of reviewed papers, including all tested parameters and means of addressing possible medication effects

Gene/condition	Participants of interest	Controls	rMT	Input‐output curve	CSP	TS definition	SICI/ICF (ISI; CS intensity)	SICF (ISI; CS intensity)	LICI (ISI; CS intensity)	Medications and other comments
*GABRG2* R43Q^24^	14 (7 with previous history of epilepsy or febrile seizures but all in remission)	24 healthy unrelated 4 non‐carrier family members	ND	NT	ND (120% of silent period threshold)	120% of rMT	↓SICI (2‐5 ms) ↑ICF (6‐15 ms) (2, 3, 4, 5, 6, 7, 10, 15 ms; 80% of rMT)	NT	NT	No participants were on medication at time of testing. ﻿All subjects underwent urine screening for use of benzodiazepines.
*GRIN1* and *GRIN2B* SNPs^26^	Total 77 healthy individuals genotyped *GRIN1*: ‐rs4880213 TT 21.4% ‐rs6293 GG 7.5% *GRIN2B*: ‐rs7301328 CC 11.3% ‐rs3764028 AA 0% ‐rs1805247 GG 0%	GRIN1: ‐rs4880213 CC 28.5%, CT 50% ‐ rs6293 AA 44%, AG 46.4% GRIN2B: ‐rs7301328 GG 40.2%, CG 48.5% ‐rs3764028 CC 94.5%, CA 5.1% ‐rs1805247 AA 87.6%, AG 12.3%	ND	NT	NT	1 mV	rs4880213 TT: SICI 2 ms, 3 ms ↓ compared to other types rs1805247 AG: ICF 15 ms ↑ compared to AA rs6293, rs7301328 rs3764028 ND (2, 3, 10, 15 ms; CS 80% of aMT)	ND (1.5, 2.1, 2.7, 3.7, 4.5 ms; CS 90% of rMT)	ND (50, 100, 150 ms; CS 120% of rMT)	The same 77 individuals were typed for all SNPs For rs1805247, in AG compared to AA, iTBS resulted in increased MEP amplitude suggestive of increased LTP‐like plasticity All participants were medication‐free at time of testing.
*STX1B* ^30^	9 (8 with previous history of epilepsy/febrile seizures).	10 healthy (age‐matched)	ND	ND (90%–140% of rMT)	NT	1 mV	ND (2, 10 ms; CS 60%–120% of rMT)	NT	NT	All participants were free of CNS‐active drugs. The minimum time off AEDs among those with previous seizures was 6 y.
*TRPV1* rs222749; rs222747^31^	rs222749: 5 TT rs222747: 12 GG All healthy adults	rs222749: 58 CC, 14 CT rs222747: 30 CC, 15 CG	ND	ND (90%–150% of rMT)	NT	Not reported	ND (2, 3, 10, 15 ms; CS 80% of aMT)	rs222749: ND rs222747: ↑for 1.5 and 2.7 ms in GG compared to CC/CG (1.5, 2.1, 2.7, 3.7, 4.5 ms; CS 90% of rMT)	ND (50, 100, 150 ms; CS 12‐% of rMT)	The same 77 individuals were typed for both SNPs. Participants’ use of drugs not explicitly stated; all reported to be healthy.
*ATP1A3‐*related AHC^36^	9 (8 with confirmed *ATP1A3* mutations); 8 competed testing	10 people with other epilepsies 10 healthy	7 patients tested between attacks: ↓compared to both control groups	3 of 5 patients showed significant intrasession variability not seen in controls (80%–120% of rMT)	NT	110% of rMT	ND (2, 10 ms; CS 80% of rMT)	NT	ND (100 ms; 110% of rMT)	One patient was tested during a hemiplegic attack ‐ motor response was elicited on either side with intensities up to 80% MSO. A second patient developed a hemiplegic attack during the testing; responses to a suprathreshold stimulus were decremental until no response was obtained. 7 of 9 people with AHC took either an AED or flunarizine whereas all epilepsy controls took AEDs. The drugs used by the patient groups did not match. The number of all drugs did not differ significantly between patient groups (people with AHC mean 2.1/person and epilepsy controls mean 2.5/person). Healthy controls took no medication.
*SCN1A‐* Dravet syndrome^41^	6 adults (5 completed protocol)	10 people with other epilepsies 10 healthy controls	ND	NT	NT	110% of rMT	↓compared to both control groups at both 2 and 5 ms (2, 5, 10, 15 ms; CS 80% of rMT)	NT	ND (100, 150, 200, 250 ms; CS 110% of rMT)	All patient participants took AEDs. The drugs used by the patient groups were not matched. The number of drugs did not differ significantly between patient groups (people with DS mean 3.2/person and epilepsy controls mean 2.5/person). Healthy controls took no medication.
*SCN1A* rs3812718^46^	49 AA	43 GG	ND	NT	Baseline: ND After CBZ: ↑ increase in AA (110% rMT)	1.5 mV	ND (3, 10 ms; CS 75% of rMT)	NT	NT	Baseline measurements were measured twice, at least 2 weeks apart, and averaged. No participants took neuroactive medications at baseline. CBZ given as a single dose of 400 mg.
*CSTB* ^52^	24	24 age‐ and sex matched	↑	NT	In patients > age 32, CSP ↑ (120% rMT)	NT	NT	NT	NT	All patients were on VPA‐based AED polytherapy; other common drugs were LEV, CZP, PRC, LTG and TPM. The number of drugs/patient was 2‐4. Healthy controls were medication free. Difference in CSP between older patients and older controls interpreted as loss of the expected age‐related shortening of CSP
*CSTB* ^53^	70	40	↑	NT	↑ (120% rMT)	NT	NT	NT	NT	All patients were on AEDs, including VPA, CZP, LEV, TPM. LTG,PRC, Healthy controls were medication free. Possible overlap with previous report^52^
*CSTB* ^57^	5 compound heterozygotes (due to high threshold, experiments only completed in 3)	24 previously reported EPM1 patients with biallelic mutations 65 healthy controls	°	NT	* (120% rMT)	NT	NT	NT	NT	° Results of individual heterozygotes compared to Z‐scores of control groups: 1 had increased rMT compared to healthy controls (in at least one hemisphere) *Results of individual heterozygotes compared to Z‐scores of control groups: 1 had increased CSP compared with biallelic patients and 2 compared with healthy controls (in at least one hemisphere) All compound heterozygous patients were on VPA‐based AED polytherapy; other drugs were LEV, CZP, PRC, LTG and TPM. The number of drugs/patient was 2‐4. See 24 biallelic patients’ AEDs two rows above.^27^ Healthy controls were medication free. The differences in TMS parameters between the two patient groups were thought to be unlikely to be explained by drugs given the similarities in drug use between these groups.
*CSTB* ^55^	10	16 healthy 5 *EPM2B*	ND	NT	NT	120% of rMT	↓SICI (across ISIs 1‐5 ms) compared to healthy controls (1, 2, 3, 4, 5, 6, 10, 15 ms; 90% of aMT)	NT	ND 30, 40, 60, 80, 100 ms; 120% of rMT	All patients were on AEDs. These were not separately reported for EPM2B and EPM1 participants. The drug status for healthy controls was not explicitly stated; presumably they were medication‐free.
*FCMTE* ^62^	6 (from same Dutch pedigree)	12 age‐ and sex matched healthy	ND	ND (100%–150% of rMT)	ND (120% of rMT)	1 mV	↓ SICI (2, 3, 4 ms) (2, 3, 4, 5, 6, 7, 10, 15 ms; 80% of rMT)	NT	NT	3 of 6 patients were on AEDs (2 AEDs/patient: 2 on VPA, 2 on CZP, 1 on CLB, 1 on OXC). Compared to controls, on visual presentation SICI appeared similarly impaired in all patients regardless of AED status. The drug status for healthy controls was not explicitly stated; presumably they were medication‐free.
*FCMTE* ^63^	4 (from same Italian family; common pericentromeric haplotype on chromosome 2)	10 age‐matched healthy	↓	Not reported	↓ (intensity not reported)	not reported	↓ (parameters not reported)	NT	NT	The drug status for patients was not explicitly stated; given they were clinically affected, presumably they may have been on AEDs. The drug status for healthy controls was not explicitly stated; presumably they were medication‐free.
*FMR1* pre‐mutation^70^	13 (70 to 170 CGG repeats)	13 age‐ and sex matched	ND	NT	ND (1 mV)	1 mV	↓2 ms (2, 3 ms; CS 80% of aMT)	NT	ND (100, 150, 200 ms; CS 110% of rMT)	Pre‐mutation carriers also had absent cerebellar inhibition, and in the SAI protocol, in contrast to controls, they lacked inhibition at ISI 20 ms All participants were free of neuroactive medication.
*FMR1* full mutation^71^	18	18 age‐ and sex matched	ND	NT	ND (1 mV)	1 mV	↓SICI (3 ms; CS 80% of rMT) ↑ICF (12 ms; CS 80% of rMT)	ND (3 ms; CS 80% of rMT)	↑ (100 ms; CS intensity same as for test)	Seven patients were on psychoactive drugs, including SSRIs and antipsychotics. No patient took AEDs. Reduction in SICI and increase in ICF remained significant even when only non‐medicated individuals were included (n = 11). All control participants were medication‐free.
*EPM2B* ^55^	5	16 healthy 10 *EPM1*	ND	NT	NT	120% of rMT	↓SICI (across ISIs 1‐5 ms) compared to healthy controls SICI at 6 ms only seen in EPM2 ↓ICF at 10 ms in EPM2 compared to controls (1, 2, 3, 4, 5, 6, 10, 15 ms; 90% of aMT)	NT	↓ 80‐100 ms in EPM2 compared to healthy controls 30, 40, 60, 80, 100 ms; 120% of rMT	All patients were on AEDs. These were not separately reported for EPM2B and EPM1 participants. The drug status for healthy controls was not explicitly stated; presumably they were medication‐free.
*NEU1* ^75^	12	12 age‐matched	ND	↑MEP at 120% (100%–120% of rMT)	↓ (120% rMT)	1 mV	↓ SICI (average 2‐4 ms)(2, 3, 4, 7, 10, 12 ms; CS 80% of aMT)	NT	NT	All 12 patients were on medication – 1 on propranolol and 11 on AEDs (all 11 on CBZ + PRC; 9 also on VPA). Patients underwent 24 h drug withdrawal before TMS. The drug status for healthy controls was not explicitly stated; presumably they were medication‐free.
*NF1* ^80^	10 (all clinically diagnosed, no history of epilepsy)	11 age‐matched	ND	NT	NT	1 mV	↑SICI (both at each ISI and averaged) (2, 3, 5 ms; CS 60% of rMT)	NT	NT	PAS with ISI 25 ms resulted in less MEP facilitation in patients compared to controls. Patients were then randomised to receive a 4‐day course of lovastatin or placebo: lovastatin was associated with normalisation of SICI and PAS 25 response (up to 45 min) No participants took neuroactive medications at baseline.
Prader‐Willi syndrome^82^	21 (13 with deletion, 8 with UDP)	11 age‐ and sex matched	↑	NT	ND (150% rMT)	120% rMT	↓ICF ↓SICI with deletion compared to UPD: SICI: average of 2, 3 ms ICF: average of 14, 16 ms CS 80% of rMT)	NT	NT	No patient took neuroactive drugs. Although not explicitly stated, it is presumed that also the healthy controls took no neuroactive medication.
*MECP2*‐ Rett syndrome^87^	17	14 age‐ and sex matched	↑	NT	↓ (140% of aMT)	N/A	NT	NT	NT	*MECP2* mutations confirmed in 14 patients, clinical diagnosis in the remaining. 13/17 patients took AEDs. Among them, 6 took only one AED while others took a minimum of 2. VPA and LTG were commonest AEDs, each used by 5 patients. No statistically significant difference in measures between those on LTG, CBZ, or LEV (thought to most affect rMT) compared to those on other AEDs.
SSADH deficiency^90^	8 (age 10‐27); diagnosed by genetic or metabolic testing	13 parents 11 healthy adults 8 age‐matched (age 9‐27)	↑compared to parents and adults, ND to age matched	↓slope in patients and young controls compared to adults (100%–180% of rMT)	↓compared to all control groups (150% rMT)	0.8 mV, except 150% of rMT for LICI	ND 3, 10 ms; CS 75% of rMT	NT	↓ compared to all control groups (150 ms; CS 150% rMT)	3 of 8 patients were taking medication (all three an SSRI, one also CBZ and another quetiapine). None of the healthy controls had a history of use of neuroactive medication.

Note

The diagnosis was genetically confirmed in all participants of interested unless otherwise stated.

Abbreviations: CBZ, carbamazepine; CLB, clobazam; CSP, cortical silent period; CZP, clonazepam; ICF, intracortical facilitation; LICI, long‐interval intracortical inhibition; ND, no significant difference; NT, not tested; OXC, oxcarbazepine; PAS, paired associative stimulation; PRC, piracetam; SICF, short‐interval intracortical facilitation; SICI, short‐interval intracortical inhibition; SSRI, selective serotonin reuptake inhibitor; VPA, sodium valproate.

### 3.1 Synaptic neurotransmission

Conditions directly affecting release of GABA may be hypothesized to affect SICI, LICI or CSP; conditions affecting function of postsynaptic GABA_A_ receptors would be expected to alter SICI. Conditions affecting release of glutamate may be hypothesized to alter ICF or SICF.

#### 3.1.1 GABRG2

The *GABRG2* gene encodes the gamma‐2 subunit of the GABA_A_ receptor. *GABRG2* mutations have been identified in epilepsy syndromes of varying severity.^21^ The R43Q mutation is associated with childhood absence epilepsy and febrile seizures.^22^ A mouse model showed reduced expression of gamma‐2 subunits and decreased inhibitory post synaptic potentials in cortical neurons.^23^ Fourteen people with the mutation, half with a previous history of epilepsy or febrile seizures, were studied.^24^ Compared to controls, SICI was reduced in keeping with the hypothesis of altered GABA_A_ergic inhibition,^24^ suggesting that genetic impairment in GABA_A_ergic neurotransmission may be detected using TMS.^24^ ICF was also increased, which may reflect the interrelatedness between the two measures as a balance between net inhibition and excitation.

#### 3.1.2 NMDARs ‐ *GRIN1*,* GRIN2B*

NMDARs are ionotropic glutamate receptors. They consist of two glycine‐N‐serine binding GluN1 subunits, encoded by *GRIN1,* and two glutamate‐binding regulatory subunits. The four types of glutamine‐binding subunits, GluN2A‐2D, are encoded by the genes *GRIN2A‐D*.^25^ Mutations in *GRIN1*, *GRIN2A* and *GRIN2B* are implicated in epilepsy and other neurological and neurodevelopmental conditions.^25^

Mori and others studied cortical excitability in 77 healthy participants with specific single nucleotide polymorphisms (SNPs) in the *GRIN1* and *GRIN2B* genes.^26^ The SNPs were rs4880213 and rs6293 for *GRIN1* and rs7301328, rs3764028, and rs1805247 for *GRIN2B*. No association with epilepsy has been reported for any of these SNPs; rs1805247 was reported to be enriched in individuals with bipolar disorder.^27^ Participants underwent paired‐pulse TMS and intermittent theta burst stimulation (iTBS) to probe long‐term potentiation (LTP) ‐like plasticity.

For rs4880213, compared to participants homozygous for the C variant or heterozygotes, participants homozygous for the T variant (allelic frequency 11.5%) had less SICI.^26^ This could imply a skewed balance between GABA_A_ergic inhibition and glutamatergic facilitation.^26^ For *GRIN2B* rs1805247, compared to participants homozygous for the A variant, heterozygotes (AG; allelic frequency 9.7%) had significantly greater ICF at 15 ms, in keeping with enhanced NMDAR function.^26^ Furthermore, iTBS led to greater MEP amplitudes in those with genotype AG, suggesting enhanced NMDAR‐dependent plasticity.^26^ Interpretation of findings for both SNPs is limited in the absence of functional evidence for pathogenicity.

#### 3.1.3 STX1B

*STX1B* encodes syntaxin‐1B, a part of the SNARE complex, which mediates synaptic vesicle release from the presynaptic membrane.^28^ Studies on neurons from a *Stx1b* null mutant mouse suggested a crucial role for syntaxin‐1B in spontaneous and evoked fast synaptic vesicle exocytosis in glutamatergic and GABAergic synapses.^29^ Loss‐of‐function mutations in *STX1B* have been identified in patients with fever‐associated epilepsies of variable severity.^28^

Nine carriers of pathogenic *STX1B* mutations showed no differences to controls in any paired‐pulse measures.^30^ The authors concluded that the results support normal GABA_A_ergic and glutamatergic excitability in asymptomatic carriers of *STX1B* mutations, perhaps influenced by compensatory mechanisms during maturation.^30^

#### 3.1.4 TRPV1

Transient receptor potential vanilloid 1 (TRPV1) channels are non‐selective cation channels which regulate the release of glutamate, and are implicated in hippocampal LTP and long‐term depression (LTD).^31^ Increased expression of *TRPV1* has been demonstrated in glutamatergic and GABAergic neurons in hippocampal and temporal cortex specimens from patients with medial temporal lobe epilepsy and it has been proposed as a potential therapeutic target in epilepsy.^32^

Mori and others studied cortical excitability in participants with one of two particular SNPs in the *TRPV1* gene: rs222749, which is not linked to substantial changes in the expression or properties of the TRPV1 channel, and rs222747, which is associated with enhanced functionality of the channel.^31^ No differences in measures emerged between the participants with different status of rs222749.^31^ For rs222747, SICF at 1.5 ms and 2.7 ms was significantly greater in the GG group compared to wild‐type participants or heterozygotes.^31^ The results were interpreted as evidence that *TRPV1* regulates glutamatergic synaptic transmission in humans.^31^

### 3.2 Neuronal membrane excitability

Conditions affecting membrane excitability of cortical excitatory interneurons and corticospinal neurones might be expected to influence cortical excitability as measured by rMT, for example. However, conditions affecting other neuronal populations may have different effects.

#### 3.2.1 ATP1A3

Alternating hemiplegia of childhood (AHC) is a rare neurodevelopmental condition characterized by paroxysmal episodes of hemiplegia and other neurological features, as well as fixed clinical features. Over half of patients have epilepsy; other common features include intellectual disability, ataxia and movement disorders.^33^ Eighty‐five percent of patients with AHC have a mutation in the *ATP1A3* gene,^34^ which encodes the α3 subunit of the Na^+^/K^+‐^ATPase ‐ the main subunit expressed in neurons.^34^ Altered function of the Na^+^/K^+‐^ATPase may lead to changes in membrane excitability and affect other integral processes dependent on ionic gradients.^34^ Evidence for increased neuronal excitability, specifically in response to high‐frequency stimulation, was demonstrated in vitro using hippocampal neurons from a knock‐in mouse model of AHC.^35^

In seven patients with AHC tested between attacks, rMT was significantly lower compared to both healthy controls and people with epilepsy.^36^ Patients showed unusual intra‐session variability in MEP amplitudes; during attacks, no response was seen.^36^ The results were thought to suggest increased cortical excitability between attacks, and reduced cortical excitability during attacks;^36^ in keeping with the findings of the mouse model.^35^

#### 3.2.2 SCN1A

*SCN1A* encodes the alpha subunit of the type 1 voltage‐gated sodium channel (NaV 1.1).^37^ NaV1.1 is highly expressed in GABAergic interneurons, where loss of function reduces excitability and impairs phasic GABA release.^38^ Variants in *SCN1A* are associated with a wide range of epilepsy phenotypes.^37^ On the severe end of the spectrum, Dravet syndrome (DS) is an epileptic encephalopathy with onset in the first year of life.^39^ Subsequently, developmental delay becomes evident and multiple seizure types, including myoclonus, occur, often with a refractory course.^39^ Most cases are associated with de novo loss‐of‐function mutations.^40^

In a TMS study of five adults with DS, SICI was absent; the difference compared to controls was significant.^41^

Lack of SICI in DS patients was thought to reflect low sensitivity of inhibitory networks to subthreshold stimuli,^41^ in keeping with a mouse model of DS, in which the threshold for action potential generation in inhibitory interneurons was increased.^42^

An *SCN1A* splice site polymorphism (rs3812718, G>A) is associated with febrile seizures.^43^ Functionally, this variant has been shown to lead to relative overexpression of the “neonatal” copy of exon 5 in the gene (5N), compared to the “adult” exon (5A).^43^ Preclinical work suggests the relative expressions of the 5N vs 5A may affect neurophysiological properties of NaV1.1, with the 5N type displaying higher sensitivity to changing temperatures^44^ and faster recovery from inactivation.^44^, ^45^ TMS was studied in 49 healthy people homozygous for the A allele at rs3812718, and compared with 43 people with genotype GG.^46^ At baseline, there were no differences in any measures between the groups. Participants were randomised to receive a single dose of either carbamazepine (CBZ) 400 mg or placebo, with TMS performed 5 hours afterwards. Compared to genotype AA, genotype GG was associated with a higher increase in CSP duration following intake of CBZ. The authors concluded that effects on GABAergic interneuron excitability may explain differences in effect for CBZ with the AA genotype compared to wild type.^47^

Taken together, reduction of GABA_A_ergic inhibition was demonstrated in Dravet syndrome, in keeping with reduced NaV1.1 function in GABAergic interneurons in this condition.^41^ In contrast, baseline measurements did not show any differences between asymptomatic carriers of an *SCN1A* variant linked with risk of febrile seizures.^46^

### 3.3 Repeat expansions

Repeat expansions are an increasingly recognised cause of disease in genetic neurological disorders. The effects on cortical excitability are unlikely to be uniform, but rather may reflect different pathological mechanisms in different conditions.

#### 3.3.1 CSTB

Unverrich‐Lundborg disease (EPM1) is an autosomal recessive progressive myoclonic epilepsy caused by mutations in *CSTB*, which encodes cystatin B, a cysteine protease inhibitor.^48^, ^49^ The most common pathogenic mutation involves an unstable expansion of a 12‐nucleotide repeat in the promoter region of the gene, leading to reduced gene expression.^49^ Clinical features include stimulus‐sensitive myoclonus and generalised tonic‐clonic seizures, with onset between ages six to 16 years.^48^ Disease progression generally occurs within the next 5‐10 years with increased myoclonus and development of ataxia and mild cognitive decline.^48^ On neuroimaging, cortical thinning, also involving the motor cortex, has been reported.^50^

There is evidence for changes in GABAergic signalling in EPM1. Using vesicular GABA transporter (VGAT) immunohistochemistry in knock‐out mice, reduction in the density of cortical GABA terminals and in cortical thickness was demonstrated.^51^ Reduced paired‐pulse depression also suggested impaired GABA_A_ergic and GABA_B_ergic inhibition.^51^ Together, these findings were thought to be in keeping with loss of GABAergic interneurons.^51^

In a series of TMS studies of patients with biallelic *CSTB* expansion mutations, compared to controls, rMT was significantly higher in patients, thought to be influenced by patients’ AED polytherapy and/or increased scalp‐to‐cortex distance.^52^, ^53^ CSP was prolonged in patients compared to controls,^54^ implying enhanced GABA_B_ergic inhibition; this was interpreted to possibly reflect a compensatory increase in inhibition to counteract hyperexcitability.^54^ In a multivariate model, CSP duration independently predicted severity of myoclonus.^53^ The size of the longer *CSTB* expansion correlated with both rMT and CSP.^54^

In a separate study of paired‐pulse TMS in ten patients with EPM1, compared to controls, SICI was reduced.^55^

TMS‐EEG over the motor area was performed in seven individuals with biallelic *CSTB* expansion mutations.^56^ Compared to healthy controls, despite higher rMT, patients had a higher P30 amplitude.^56^ The amplitudes of N100 and P180 were lower in patients compared to controls.^56^ Compared to controls, 25‐100 ms following TMS, patients showed lower power in the alpha and beta bands over both M1s and lower gamma power over the vertex; also inter‐trial coherence in the alpha and beta bands was reduced.^56^

TMS‐EMG results were also reported for five compound heterozygous patients, all of whom had a monoallelic repeat expansion in *CSTB*, with the other allele affected by a common point mutation (c.202C>T).^57^ Thresholds appeared elevated and CSP prolonged.^57^ Despite the limitation of small sample size and limited statistical testing, the findings were thought to fit the more severe phenotype of the compound heterozygous form.^57^

In summary, SICI was reduced in keeping with impaired GABA_A_ergic inhibition.^55^ Prolonged CSP implies enhanced GABA_B_ inhibition and the degree of prolongation may correlate with genotypic and phenotypic severity. However, lower amplitudes of N100 and P180 in EPM1^56^ may point towards the opposite ‐ decreased GABA_B_ inhibition (Table 2). The authors suggested that in contrast to impaired baseline cortico‐cortical inhibition captured by TEPs, prolonged CSP could reflect excessive cortico‐spinal inhibition.^56^ The difference might also relate to the fact that CSP is elicited during volitional activity whereas the TEP is elicited at rest.

**TABLE 2:** Summary of TMS findings in EPM1 and EPM2

TMS parameter	Presumed GABA involvement	Findings in EPM1	Findings in EPM2
TMS‐EMG SICI	GABA_A_	Impaired^55^	Impaired^55^
TMS‐EMG CSP	GABA_B_	Enhanced^52^, ^53^	Not tested
TMS‐EMG LICI	GABA_B_	No difference to controls^55^	Impaired^55^
TMS‐EEG N100/P180	GABA_B_	Impaired^56^	Not tested

Abbreviations: CSP, cortical silent period; LICI, long‐interval intracortical inhibition; SICI, short‐interval intracortical inhibition.

#### 3.3.2 Familial cortical myoclonic tremor with epilepsy

Familial cortical myoclonic tremor with epilepsy (FCMTE), also known as familial adult myoclonic epilepsy (FAME), autosomal dominant cortical myoclonus and epilepsy (ADCME), and benign adult familial myoclonic epilepsy (BAFME), is characterized by myoclonus affecting the limbs distally and generalized tonic‐clonic seizures, with onset in the second or third decade.^58^ Additional features may include mild cerebellar signs and cognitive decline.^58^ The inheritance pattern is autosomal dominant and, recently, inserted pentamer repeats, presumed to lead to RNA toxicity, have been identified in *STARD7*,* SAMD12*,* TNRC6A* and *RAPGEF2*,^59^, ^60^, ^61^ in keeping with previously implicated loci.^58^

Compared to controls, six members of a Dutch FCMTE pedigree had reduced SICI at ISIs 2 and 3 ms.^62^ Compared to controls, four members of an Italian family affected with FCMTE were reported to have a significantly lower rMT, reduced duration of CSP, and reduced SICI.^63^

Findings of reduced SICI in both studies suggest impaired GABA_A_ergic inhibition, also seen in other myoclonic epilepsies. It would be interesting to explore possible changes in GABA_B_ergic inhibition, implied by reduced duration of CSP, using LICI.

#### 3.3.3 FMR1

Fragile X‐related disorders are associated with excess repeats of the CGG trinucleotide in the promoter region of the *FMR1* gene. The product of this gene is the fragile X mental retardation protein 1 (FMRP), an RNA‐binding protein with high brain expression levels. A CGG repeat number of over 200 is associated with epigenetic silencing of the *FMR1* gene.^64^ This is termed the full mutation and is associated with Fragile X syndrome (FXS), characterized by language delay, hyperactivity, intellectual disability, anxiety and certain physical features.^64^ Seizures occur in 3%–16%.^64^

Fragile X premutation involves 50‐200 excess CGG repeats and is associated with RNA gain‐of‐function toxicity.^65^ Individuals may present with varying phenotypes including affective disorders, ADHD and primary ovarian insufficiency. A subset may develop fragile X‐associated tremor/ataxia syndrome (FXTAS) in later life, characterized by intention tremor, cerebellar ataxia, cognitive decline and Parkinsonism.^65^ Neuroimaging features include cerebellar and brain stem atrophy and white matter changes, some of which may be present also in asymptomatic carriers.^65^, ^66^

Excessive glutamate‐mediated signalling via metabotropic group I receptors (mGluRI) is among presumed disease mechanisms.^64^ Altered neuroplasticity and reduced expression of GABA_A_ receptor subunits have been demonstrated in animal models of the full mutation^64^; tonic, but not phasic, inhibition was found to be altered.^67^ The picture may be further complicated by changes in expression of proteins involved in GABA metabolism.^68^ In the premutation, findings regarding GABA have been less consistent. Increased GABA_A_ inhibition was shown in the cerebellum^68^; another study demonstrated that an abnormal firing pattern in hippocampal neurons was rescued by the GABA_A_ agonist allopregnanolone, implying an underlying impairment in GABA_A_ergic inhibition.^69^

Thirteen asymptomatic women harboring the Fragile X premutation had significantly less SICI at 2 ms compared to controls.^70^ The authors concluded that the differences in mutation carriers and controls were in keeping with GABA_A_ergic dysfunction with relatively preserved GABA_B_ function.^70^

In a study of 18 individuals with Fragile X, compared to controls, patients had reduced SICI and increased ICF and LICI.^71^ The findings were interpreted as evidence for reduced GABA_A_ inhibition, along with excessive glutamatergic signalling, as a disease mechanism.^71^ Increased LICI was thought to imply preserved postsynaptic GABA_B_ inhibition.^71^

### 3.4 Other mechanisms

#### 3.4.1 EPM2B

Lafora body disease (EPM2) is another progressive myoclonic epilepsy with autosomal recessive inheritance.^72^ It typically presents in adolescence with stimulus‐sensitive myoclonus with a rapidly progressive and fatal disease course.^72^ Most cases of EPM2 are caused by mutations in the *EPM2A* or *EPM2B* genes, which lead to abnormalities in the regulation of glycogen metabolism and polyglucosan accumulations (Lafora bodies).^72^ In a mouse model of EPM2A, Lafora bodies first appeared in GABAergic neurons, the number of which was reduced compared to wild type prior to the development of the inclusions.^73^

Canafoglia and coworkers studied paired‐pulse TMS in ten patients with EPM1 and five patients with EPM2.^55^ At ISIs 1‐5 ms, SICI was significantly reduced in patients compared to controls with no difference between EPM1 and EPM2.^55^ Compared to controls, EPM2 patients showed inhibition instead of facilitation at 10 ms; they also had significantly less LICI at 80 ms and 100 ms.^55^ In summary, the findings implied impaired GABA_A_‐ and GABA_B_‐mediated inhibition in EPM2B. The finding of reduced ICF was suggested to reflect a compensatory phenomenon to counteract epileptic activity.^55^

#### 3.4.2 NEU1

Sialidosis is an autosomal recessive lysosomal storage disorder caused by mutations in the *NEU1* gene, which encodes a lysosomal neuraminidase.^74^ Type I sialidosis is a progressive myoclonic epilepsy with onset in the second or third decade; characteristics include macular changes known as ‘cherry‐red spots’ and ataxia.^74^

Compared to controls, 12 individuals with sialidosis I had greater MEP amplitudes, while SICI and CSP duration were significantly reduced.^75^ The findings were thought to be in keeping with increased excitability and reduced GABA_A_ergic, and possibly also GABA_B_ergic, inhibition.^75^

#### 3.4.3 NF1

Neurofibromatosis I is an autosomal dominant disorder caused by loss‐of‐function mutations in the *NF1* gene encoding neurofibromin 1, an inhibitor of the RAS pathway.^76^ Characteristic features include neurofibromata, multiple café‐au‐lait macules, skinfold freckling, Lisch nodules, and certain skeletal abnormalities and malignancies.^76^ Epilepsy occurs in 14%.^77^

A knock‐out mouse model of NF1 showed increased RAS‐dependent GABA release and impairment of LTP and learning.^78^, ^79^ In a study of ten people with clinically diagnosed NF1, SICI was significantly increased compared to controls.^80^ Paired‐associative stimulation (PAS) suggested impaired cortical LTP‐like plasticity.^80^ Patients were randomized to a four‐day course of either lovastatin, an inhibitor of the RAS pathway, or placebo. Compared to those receiving placebo, patients who received lovastatin showed normalisation of SICI; lovastatin but not placebo was also associated with PAS‐associated MEP facilitation.^80^ The findings were thought to imply that reduced LTP in NF1 may be mediated by increased (GABA_A_ergic) cortical inhibition.^80^

#### 3.4.4 Prader‐Willi syndrome

Prader‐Willi syndrome (PWS) is characterized by infantile hypotonia, developmental delay, excessive eating, behavioural problems, hypogonadism, short stature, and characteristic facial features; seizures are present in 10%–20%.^81^ PWS is caused by lack of expression of the paternally‐derived copies of genes in the region 15q11.2‐q13, which normally constitute, due to genomic imprinting, the active copies of the genes.^81^ This is most often due to either deletions involving the paternally‐inherited chromosome, or maternal uniparental disomy (UDP).^81^

Civardi and others studied 21 patients with PWS, of whom 13 had a deletion and 8 had UPD.^82^ Compared to controls, patients had significantly higher rMT and reduced ICF.^82^ SICI was significantly decreased in patients with a deletion compared to those with UDP.^82^

The 15q11‐q13 region contains 50‐100 genes, including genes encoding subunits of the GABA_A_ receptor; reduced expression of *GABRA5* and *GABRB3* was demonstrated in lymphoblastoid cells from individuals with PWS.^83^ On the other hand, increased expression *GABRA4* and *GABRG2*, which encodes the gamma‐2 subunit common in postsynaptic GABA_A_ receptors, was also shown.^83^ The possible effects of these changes on synaptic GABA_A_ergic transmission are unclear.

Decreased SICI in patients with deletions compared to patients with UDP implies lower GABA_A_ergic inhibition in the deletion subgroup. It was noted that the incidence of seizures in PWS patients with deletions is higher compared to those with UDP.^82^ However, in a previous study of gene expression in PWS patients, no differences in expression of GABA‐related genes were found between patients with deletions and those with UPD.^83^

Reduced ICF was contrary to the authors' hypothesis.^82^ It was postulated that the actual observations might reflect altered inhibition/excitation balance due to overstimulation of GABA_A_Rs not affected by the mutation;^82^ this needs further elucidation.

#### 3.4.5 Rett syndrome

Rett syndrome is a disorder characterized by developmental regression, including early loss of language and hand skills, gait abnormalities, and stereotypical hand movements; epilepsy is also common.^84^ Most patients are female and the majority of typical Rett cases are associated with mutations in the X‐linked *MECP2* (methyl‐CpG‐binding protein‐2) gene. The protein product, MeCP2, regulates gene expression and is especially abundant in neurons; its function appears integral for normal brain function throughout life.^84^ Reduced expression of *GABRB3* and *UBE3A*, which encodes a ubiquitin protein ligase involved in proteostasis in the brain, have been demonstrated.^85^ In a mouse model, lack of expression of *Mecp2* in GABAergic neurons was sufficient for reproducing many characteristics of Rett syndrome, including electrographic seizures.^86^

In a single‐pulse TMS study of seventeen patients with Rett syndrome, motor thresholds were significantly elevated compared to controls, interpreted to reflect reduced brain volume and abnormal cortico‐cortical connections.^87^ CSP duration was reduced compared to controls, suggesting reduced GABA_B_ergic inhibition^87^; it would be interesting to also study SICI and LICI to explore this further.

#### 3.4.6 SSADH deficiency

Succinic semialdehydase (SSADH) deficiency is an autosomal recessive disorder caused by mutations in the *ALDH5A1* gene leading to impaired gamma‐aminobutyric acid (GABA) degradation and, in turn, to accumulation of gamma‐hydroxybutyric acid (GHB) and GABA.^88^ Other metabolic changes, including oxidative stress and dysregulation of autophagy may have a role in the pathogenesis of SSADH deficiency.^88^, ^89^ Characteristic features of SSADH deficiency include developmental delay with emphasis on language skills, hypotonia, ataxia, seizures, and behavioural problems.^88^

Eight patients with SSADH deficiency were studied and results compared to those of parents (obligate heterozygotes), and healthy controls.^90^ CSP was significantly shorter in patients compared to all control groups; also LICI was nearly absent in patients but present in all control groups.^90^ It was postulated that downregulation of postsynaptic GABA_B_ receptors, which has been demonstrated in an animal model of SSADH deficiency, could lead to increased binding of GABA to presynaptic GABA_B_ receptors and reduced activity‐dependent secretion of GABA.^90^ Supporting this theory, previous PET imaging studies had shown reduced benzodiazepine binding in SSADH. In the fetal brain, GABA exerts a depolarising effect due to reversed directionality of chloride transport associated with GABA_A_‐receptor activation.^89^ The authors postulated that exposure to high levels of GABA during this time could trigger compensatory mechanisms, with unpredictable effects on later GABAergic balance.^90^

## 4 DISCUSSION

**FIGURE 4:**
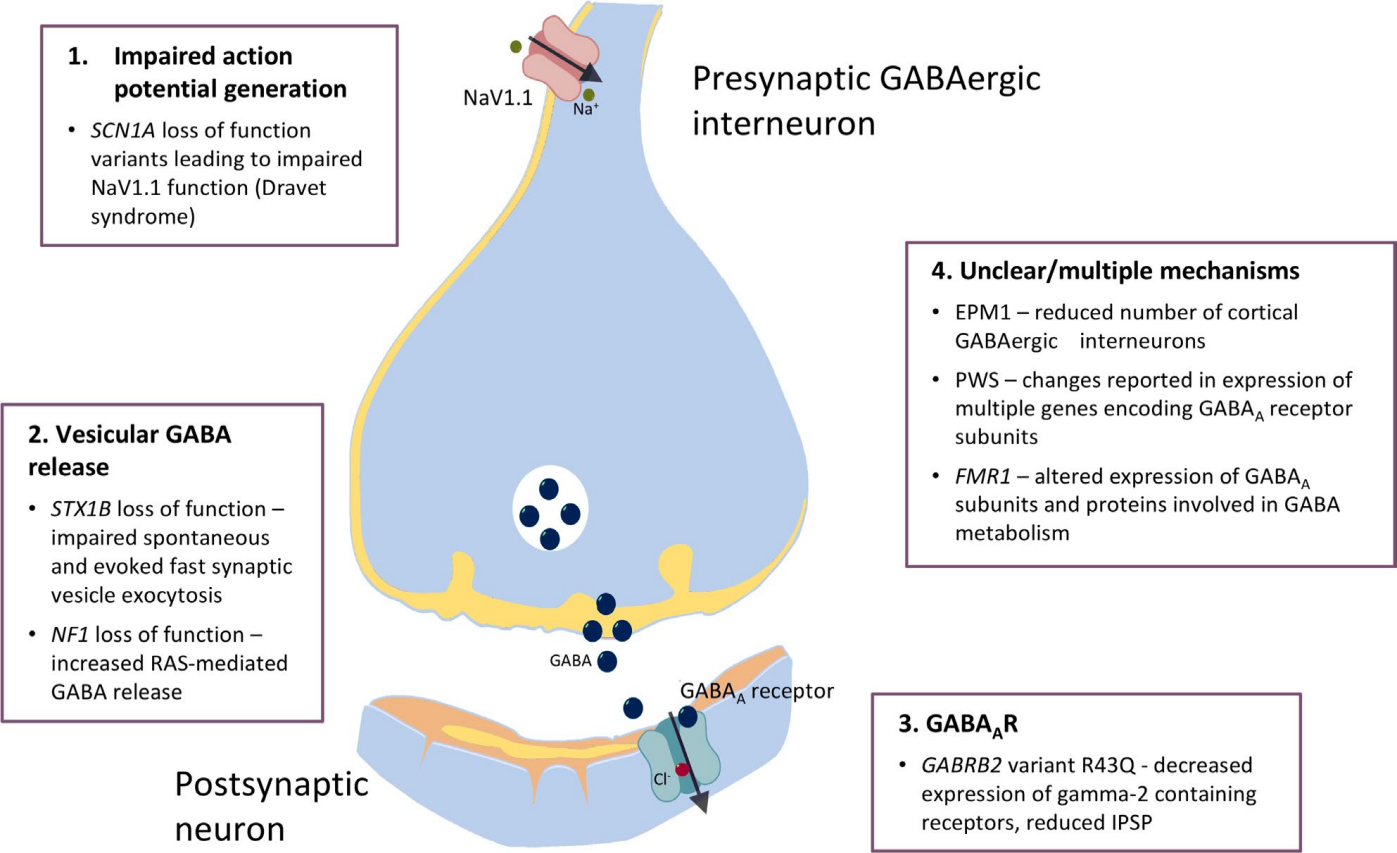
Summary of impact of some conditions reviewed in this paper on synaptic GABA_A_ergic signalling. These may be grouped into: 1. Impaired excitability of presynaptic GABAergic interneurons; 2. Altered release of GABA from presynaptic terminals; 3. Altered function of postsynaptic GABA_A_ receptors. 4. For several conditions, there is evidence for altered GABA activity/levels, but how these impact on synaptic function is unclear.

We have presented several examples of epilepsy‐related genetic conditions with evidence of altered inhibition or facilitation as measured by TMS. For many, findings are congruent with hypotheses of the effects of the conditions/variants on the GABA system, particularly synaptic GABA_A_ergic transmission (Figure 4). This is most evident with reduced SICI in individuals with loss‐of‐function variants in *GABRG2*,^24^ and in individuals with *SCN1A‐*related Dravet syndrome.^41^

### 4.1 Decreased GABA_A_ergic inhibition in myoclonic epilepsies

Decreased GABA_A_ergic SICI was demonstrated in both EPM1 and EPM2,^55^ consistent with existing evidence of loss of GABAergic neurons.^51^ Reduced LICI in EPM2 suggests impaired GABA_B_ inhibition.^55^ In EPM1, prolonged CSP implies enhanced GABA_B_ inhibition but lower amplitudes of TEP components N100 and P180 might suggest the opposite.^56^ What these different parameters tell us about alteration of GABA_B_ergic inhibition in EPM1 should be studied further.

In EPM1, the TMS‐EEG power spectra changes and altered inter‐trial coherence were thought to reflect impaired function of cortico‐cortical/cortico‐subcortical motor circuits.^56^ In healthy people, the effects of GABA_A_ergic drugs on TEP components were sometimes seen only on the contralateral hemisphere, suggesting that changes in GABA_A_ergic inhibition may be associated with altered thalamocortical connectivity.^91^ From this perspective, it would be interesting to study, with TMS‐EEG, the connectivity patterns in EPM1 further, and correlate these with the other GABAergic parameters.

EPM1 has emerged as a particularly informative condition also due to the genotype‐TMS correlation that emerged: the size of the expansion mutation correlated with rMT and CSP duration.^53^ Further, CSP correlated with phenotypic severity as measured by the degree of myoclonus.^53^ These findings show promise for use of TMS biomarkers in research and clinical practice.

GABAergic inhibition was also impaired in familial cortical myoclonic tremor with epilepsy,^62^, ^63^ and another progressive myoclonic epilepsy, sialidosis type 1.^75^ Within the literature on TMS in epilepsies without confirmed (monogenic) genetic etiology, juvenile myoclonic epilepsy and benign myoclonic epilepsy are associated with impaired SICI.^92^, ^93^ Indeed, TMS may be particularly suited for studying myoclonic epilepsies.

There is evidence for the role of GABA_A_ergic mechanisms in myoclonus of other etiologies than EPM1: in rats, intraventricular injection of a GABA_A_ antagonist precipitated myoclonus.^94^ The efficacy of GABA_A_ergic drugs such as benzodiazepines in the treatment of myoclonus of various etiologies^95^ may further support the role of GABA_A_ impairment in the pathogenesis. In a single report of a patient with focal epilepsy of unknown etiology, whose seizures included negative myoclonus involving the right upper limb, [^123^I]iomazenil single photon emission computed tomography suggested reduced GABA_A_ function in the left medial frontal area.^96^ As such, the findings of impaired GABA_A_ergic transmission in epilepsies with myoclonus are plausible.

### 4.2 Sources of ambiguity

For some conditions and findings, synthesising TMS results with pathophysiology was not straightforward. In SSADH deficiency, contrary to the authors’ hypothesis in a condition with elevated levels of GABA, TMS findings suggested *reduced* GABA_B_ activity.^90^ The authors postulated that this could arise due to a negative feedback loop, or through some kind of compensatory mechanism. There is a risk of circularity if among a multitude of possible mechanisms, the one that fits the results may be chosen with no attempt to substantiate the interpretation. In reviewing reports of conditions with TMS evidence for altered GABA_A_ activity, it is useful to consider whether reported data on subunit expression are compatible with synaptic or extrasynaptic changes. The GABAergic system is complex and remains a focus of research; used optimally, TMS could contribute to elucidating GABAergic changes in disease. Overall, with expanding use of these measures, and previously voiced concerns,^97^ it would be prudent to establish community standards for protocols and quality control.

Although the dependence of SICI on GABA_A_ergic inhibition is well‐established, SICI may, particularly with higher CS intensities, be contaminated by SICF. Therefore, findings of reduced SICI could be influenced by increased glutamatergic facilitation captured by SICF.^98^ However, in the case of minor allele homozygotes for the *GRIN1* SNP rs4880213, reduced SICI did not appear to be explained by increased SICF.^26^ As the optimal ISIs for inducing SICF may differ between individuals, experimental confirmation of abnormal SICI would ideally include selecting ISIs based on individual SICF and applying multiple CS intensities.^98^ This may be challenging in some patient groups, but would be particularly valuable where unexpectedly impaired SICI was observed.

In contrast to studies in patients or mutation carriers, in two studies TMS parameters were correlated with genotype at polymorphic sites. For *TRPV1*, homozygotes for a SNP linked with enhanced channel functionality showed increased SICF, whereas for another SNP not known to influence channel function, no differences according to genotype were seen.^31^ This could present a functionally relevant TMS signature. In contrast, the results of the study on *GRIN1* and *GRIN2B* SNPs^26^ warrant some caution in interpretation: all genotypes were relatively common, without a clear disease or functional association. These observations emphasize that studies should be carefully designed with both biologically plausible hypotheses and clinically relevant phenotypes, whether disease‐ or trait‐related.

### 4.3 Challenges and limitations

A recognised problem in studies of brain stimulation is inter‐trial variability.^99^ Achieving large study size is limited by the rarity of these conditions, as well as practical challenges in conducting experiments in individuals affected by severe epilepsies. These same issues pertain to ensuring sufficient power to detect possible changes. Examples from this review^41^ support the notion that robust changes may be detected even with small sample size; it would be important to confirm findings in sufficient numbers of patients.

Especially for studies of SNPs with relatively high allelic frequencies, care in experimental design should be reflected in choice of participants. In the study of *GRIN1* and *GRIN2B* SNPs, the sample included no minor allele homozygotes, likely to reduce power.^26^ In both of the SNP studies, the same participants were genotyped for the all SNPs and all patients were included in comparisons across a particular SNP regardless of type of other SNPs. If all SNPs were assumed to potentially exert an effect of similar magnitude on NMDAR‐mediated neurotransmission, this approach may have undermined ability to detect significant effects: more sophisticated models may be needed.

Even with sound hypotheses, not all reviewed studies identified TMS changes. *STX1B* is an emerging epilepsy gene; mutations are expected to affect synaptic function. However, carriers of mutations in this gene, most of whom had a history of seizures providing some evidence of pathogenicity, failed to show evidence of altered GABA or glutamatergic neurotransmission as measured by TMS‐EMG.^30^ This could reflect age‐dependency of the disease process, but could also be due to the number of limitations of the technique.

An obvious limitation of TMS‐EMG is that it only samples the motor cortex. Although the germline variants are present in all DNA of an individual, there are regional differences in gene expression in the brain. Normal patterns, for example of GABA inhibition, demonstrated by TMS‐EMG do not rule out abnormalities in GABAergic activity within other networks or regions. In these respects, TMS‐EEG may offer advantages. Further, this technique may allow use of intensities lower than those required for TMS‐EMG,^16^ which may allow extension of studies to more individuals in conditions where stimulator output was insufficient to evoke motor responses in all participants.^36^, ^57^ Compared to TMS‐EMG, the neurophysiological underpinnings of TMS‐EEG measures are less well defined and methodology less standardised. The influence of peripheral evoked potentials on TEPs remains an important concern,^18^, ^19^ and there is consensus for the need to better understand and mitigate such confounding effects.^100^, ^101^ Addressing these issues will be important for possible future biomarker use.

Some genetic epilepsies, such as EPM1,^53^ are associated with cortical atrophy. A linear correlation between scalp‐to‐cortex distance and rMT has been reported^102^; as conditioning stimulus intensities are generally chosen as a percentage of rMT, one would not expect the relative stimulus intensities of conditioning stimuli to differ between individuals with different scalp‐to‐cortex distances. However, modelling has shown that eg sulcal widening may lead to alterations in the current density distributions, which may lead to differences in the populations of neurons targeted,^103^ and possible effects of anatomical changes must be considered.

The possible confounding effect of medications, particularly AEDs, is a further limitation and must be considered in the interpretation of findings (Table 1, Table S1). One would not expect the GABA_A_ergic effects of benzodiazepines to lead to erroneous findings of impaired SICI, but the mechanisms of action for many AEDs are incompletely characterised. Although studying unaffected carriers or patients in remission could be advantageous in this regard, such cases may not be fully representative of the condition of interest, as exemplified by the normal findings in asymptomatic carriers of *STX1B*.^30^ The issue of confounding effects of AEDs can be addressed to some extent by recruiting controls with epilepsy, taking similar medications to the group of interest, although medications may be difficult to match completely.^41^ Participants may also be stratified by drug status^71^; small sample size is a caveat. In one study, potential medication effects were countered by means of withdrawing antiepileptic medication for a minimum of 24 hours.^75^ In addition to being generally of some concern due to the risk posed to patients, this may be unlikely to offset possible medication effects on measures of interest.

### 4.4 TMS ‐ one tool among others

It is important that studies are hypothesis‐driven, and avoid circularity of arguments. Among the conditions discussed in this review, similar changes in TMS parameters are described for very different conditions. The notion that pathophysiological specificity of TMS may be overestimated is exemplified by findings of not only decreased SICI, but also increased ICF in people with the *GABRG2* variant R43Q. Indeed, any use of TMS for diagnostic purposes would require integration with other investigations. As such, it could be valuable in certain scenarios. When patients are found to have a previously unreported variant in a disease‐implicated gene, it may not be clear whether the variant is pathogenic or not. For some genes, both loss of function and gain of function variants may be pathogenic, and the directionality of the change in function may not be apparent based on phenotype alone. Such a situation could arise, for example, in the context of a patient with severe epilepsy found to harbor an *SCN1A* mutation not previously reported, with a phenotype not fully concordant with Dravet syndrome. The ability to use an in vivo functional assessment of an entire system, using TMS, could be valuable in this situation, and have implications for therapeutic choices, such as avoiding treatment with sodium channel blockers.^38^ Further, correlations between TMS findings and genotypic or phenotypic severity, as seen in EPM1, suggest a potential role for TMS in predicting disease trajectory and/or treatment response, which should be explored further.

## 5 CONCLUSIONS

Genes associated with epilepsy are being uncovered at a rapid pace. As a result, more tools are required to study the in vivo effects of variants. Despite limitations, TMS could be useful for exploring the consequences of variants on the function of interneuron circuits. TMS‐EMG has already been successfully applied to this end in several conditions; more research is required to expand and corroborate these findings. TMS‐EEG may offer a further tool to do so, but the neurophysiological underpinnings do need to be better characterised.

## CONFLICT OF INTEREST

None of the authors has any conflict of interest to disclose. We confirm that we have read the Journal's position on issues involved in ethical publication and affirm that this report is consistent with those guidelines.

## Supporting information

Supplementary Material
