# Supplementary material for: Transcranial magnetic stimulation as a tool to understand genetic conditions associated with epilepsy
Source: Epilepsia. 2020 Aug 12;61(9):1818–39. doi: 10.1111/epi.16634 (PMC8432162; doi:10.1111/epi.16634)
Supplement: Supplementary file 1 — Supplementary Material [file EPI-61-1818-s001.docx]

**Supplementary Material for “Transcranial magnetic stimulation as a tool to understand genetic conditions associated with epilepsy”**

Katri Silvennoinen, MD, Simona Balestrini, MD, John C Rothwell, PhD, Sanjay M Sisodiya, FRCP

**BACKGROUND**

**GABA**

GABA, the major inhibitory neurotransmitter in the central nervous system, binds two types of receptors, A and B. Type A is a ligand-gated chloride channel, formed of five subunits. Receptors with a gamma subunit are mainly localised postsynaptically, where they mediate phasic inhibition through fast inhibitory postsynaptic potentials.^1^ In contrast, type A receptors with delta subunits are localised extrasynaptically and mediate tonic inhibition.^2^ The type B receptor is a metabotropic receptor; its activation leads to a slower hyperpolarization mediated via a G-protein-coupled potassium channel.^3^ Benzodiazepines, positive allosteric modulators which bind to the interface between the alpha and gamma subunits of the GABA_A_ receptor,^1^ have been extensively used to study GABAergic modulation of various TMS parameters.^4–7^

**TMS-EMG**

TMS is thought to predominantly depolarize axons of cortical interneurons, which then activate pyramidal cells transsynaptically causing a volley of action potentials termed the I1 wave to propagate down the corticospinal tract.^8^ Several subsequent volleys, termed later I-waves, occur at a periodicity of approximately 1.5ms, presumed to reflect interactions between pyramidal cells and layer II/III interneurons.^8^ Motor evoked potentials (MEPs) may be visualized on the EMG trace recorded from the corresponding muscle.^9^

#### Motor threshold and stimulation-response measurements

The resting motor threshold (rMT) is defined as the minimum stimulus intensity required to elicit MEPs of over 50µV in at least 5 out of 10 trials in the corresponding resting muscle, expressed as percentage of maximum stimulator output (MSO).^9^ The corresponding measure from contracted muscle is termed active motor threshold (aMT). Stimulation intensities lower than individual rMT are referred to as subthreshold, those higher as suprathreshold.^9^

Through their role in initiating action potentials, voltage-gated sodium channels are integral to maintenance of axonal membrane excitability.^10^ Pharmacological studies have consistently shown an increase in rMT following administration of drugs with sodium channel blocking properties, suggesting rMT reflects axonal membrane excitability.^11,12^ Glutamate is the major excitatory neurotransmitter in the brain, acting via various subtypes of both AMPA and NMDA receptors expressed in the human motor cortex, including in layers II and III.^13^ The function of ionotropic glutamatergic synapses at excitatory connections to corticospinal neurons is also postulated to influence rMT,^14^ although pharmaco-TMS evidence for this is somewhat inconsistent.^15,16^

MEP amplitude increases with stimulus intensity in a sigmoidal fashion, reflecting an increase in the number of recruited corticospinal fibres, and increased temporal synchronization.^9^ Sodium channel blockers are associated with a reduction in the slope of the stimulus response curve,^17^ as was lorazepam;^6,17^ this effect may be mediated by a reduction in the amplitude of later I-waves.^4^ There is pharmacological evidence also for glutamatergic, noradrenergic and serotonergic modulation of MEP amplitude.^14,15^

#### Cortical silent period

A suprathreshold TMS pulse causes transient cessation of voluntary motor activity known as the cortical silent period (CSP).^9^ The initial 50ms of the CSP is thought to reflect spinal inhibition, whereas the later part is considered cortical in origin.^9^ Pharmacological studies implicate GABA_B_ergic inhibition.^18–20^ GABA_A_ activation appears to also have a modulatory effect, the direction of which appears to depend on stimulation intensity;^5,6^ see Supplementary Table 1.

### Paired-pulse TMS

#### Short-interval intracortical inhibition

In short-interval intracortical inhibition (SICI), a subthreshold CS leads to inhibition of the MEP evoked by the subsequent test stimulus, most evident at ISI of 1-5ms.^21^ The intracortical origin of SICI was demonstrated in cervical epidural recordings in humans: both later I-waves and MEPs were suppressed in response to SICI conditioning.^22^ The CS, through recruitment of low-threshold inhibitory circuits, is thought to lead to inhibition of late I-waves either by inhibiting their generation or through summation of inhibitory post-synaptic potentials on cortical pyramidal neurons.^8,23^ The idea that SICI reflects phasic synaptic GABA_A_ inhibition is supported by consistent evidence that SICI at ISIs 2ms and longer is increased when classic benzodiazepines are administered.^7,14,24,25^ In contrast, SICI at short ISIs of ~1ms may reflect refractoriness of cortico-cortical axons or tonic extrasynaptic GABA_A_ activity.^26,27^ GABA_B_ergic activity may also modulate SICI through presynaptic autoinhibition of GABA release.^14,20,28^ Particularly with higher CS intensities (e.g. 90% rMT), SICI may be contaminated by short-interval-intracortical facilitation (SICF; see below).^29^ Pharmacological studies have shown that SICI is modulated by activity of other neurotransmitter systems, including noradrenaline, dopamine, and serotonin.^30^

#### Intracortical facilitation

Intracortical facilitation (ICF) occurs when the ISI is longer, typically 10 or 15ms.^21^ Due to some overlap in the ISIs which may produce either SICI or ICF, ICF is considered to represent a net effect of increased facilitation over GABA_A_-mediated inhibition, which is supported by findings of reduced ICF following administration of benzodiazepines.^31^ Despite uncertainty about its exact neural substrates, ICF appears to be mediated by an increase in NMDAR-mediated glutamatergic transmission, as it is decreased following administration of NMDAR antagonists.^32,33^

#### Long-interval intracortical inhibition

Long-interval intracortical inhibition (LICI) is elicited when a suprathreshold CS precedes the TS by 60-200ms;^34,35^ LICI was associated with reduced late I-waves in epidural recordings.^34^ LICI is attributed to ﻿GABA_B_ receptor-mediated inhibition, supported by findings of the increasing effect of a GABA_B_ agonist on LICI.^36^ The longer interstimulus interval and higher conditioning stimulus intensity required for LICI compared to SICI are in keeping with the profiles of motor cortex GABA_B_ vs GABA_A_-dependent inhibitory postsynaptic potentials.^34^

#### Short-interval intracortical facilitation

Short-interval intracortical facilitation (SICF) (Figure 2) is elicited at specific ISIs of approximately 1.1-1.5, 2.3-2.9, and 4.1-4.5ms, when a near-threshold CS follows the TS.^37^ The ISIs associated with SICF correspond to intervals observed between I-waves; SICF is thought to be caused by GABA_A_-dependent phase-specific interactions between neural populations producing the I-waves.^16,38,39^

### TMS-EEG

The TEP presents the summation of inhibitory and excitatory postsynaptic potentials. Pharmacological studies have provided some links between specific components and GABAergic activity.

The magnitude of the components varies according to the site of stimulation and the channel(s) used to measure them. Among the components (Figure 3), N45 may reflect GABA_A_ergic inhibition, as evidenced by positive modulation by benzodiazepines of the N45 in the hemisphere contralateral to stimulation.^45^ In contrast, the magnitude of N100 may reflect GABA_B_ergic inhibition, as evidenced by positive modulation by a GABA_B_ agonist on the N100 ipsilateral to stimulation.^45^

The origin of P180 has been less well defined, and it is recognised that it may be influenced by auditory activity evoked by the stimulus click.^46^ A LICI protocol suppressed P180; the suppression was enhanced by a GABA_B_ agonist,^47^ suggesting that this potential reflects function of intracortical circuits modulated by GABA. There is some correlation between measures derived from TMS-EMG and TMS-EEG, e.g. the N15-P30 component evoked by stimulation of M1 and measured from channels around the stimulation site is correlated with simultaneously measured MEP amplitude.^48^ However, MEPs and TEPs have been shown to be influenced differently by alterations in pulse characteristics.^49^ Indeed, while the neuronal populations whose response is sampled by TMS-EMG and EEG are overlapping, they are partly different (e.g. TMS-EMG samples the response of the entire corticospinal tract and lower motor neuron, whereas TMS-EEG samples the response of cortical areas connected through corticothalamic and other circuits). Hence, measures from the two modalities are unlikely to be directly comparable.

Despite these advances, the understanding of the neurophysiological underpinnings and the factors contributing to TEP components is incomplete. Studies employing auditory and somatosensory sham have shown that TEP components may be influenced by auditory and somatosensory evoked potentials.^40,41^ Although later components (N100-P180) may be particularly affected, also earlier components including P60 may be involved.^40–42^ Consequently, there is a drive to appraise reproducibility and to standardise experimental procedures and data processing.^43,44^

**METHODS**

**Literature search**

The search was limited to studies describing data from humans published in English up to 31/12/2019. The 768 results were screened by reviewing the abstract or, as appropriate, title only. The majority were excluded for the following reasons: publications not involving original research, work not related to TMS, preclinical work, reports of physiological phenomena in healthy and non-genotyped people, methodology/safety reports, studies of non-genetic epilepsy, or of other non-genetic neurological or psychiatric conditions, and studies of repetitive TMS or other plasticity protocols. Of the remaining, 32 were relevant for conditions or genes of potential interest but did not report findings of intracortical facilitatory or inhibitory phenomena, or TMS-EEG, and were therefore not deemed relevant for the review. The conditions/genes implicated in the remaining publications were reviewed for relevance to epilepsy. Finally, 23 publications on 15 different genes/conditions were included.

**SUPPLEMENTARY REFERENCES**

1. Cherubini E. Phasic GABAA-Mediated Inhibition. In: Noebels JL, editor. Jasper’s Basic Mechanisms of the Epilepsies. 4th ed. Oxford University Press; 2012. p. 97–110.

2. Walker MC, Kullman DM. Tonic GABAA receptor-mediated signaling in epilepsy. In: Noebels JL (Ed) Jasper’s Basic Mechanisms of the Epilepsies. 4th Ed. Oxford: Oxford University Press, 2012:147–62.

3. Magloire V, Mercier MS, Kullmann DM, Pavlov I. GABAergic Interneurons in Seizures: Investigating Causality With Optogenetics. Neuroscientist 2019;25:344–358.

4. Di Lazzaro V, Oliviero A, Meglio M, Cioni B, Tamburrini G, Tonali P, et al. Direct demonstration of the effect of lorazepam on the excitability of the human motor cortex. Clin Neurophysiol 2000;111:794–9.

5. Ziemann U, Lönnecker S, Steinhoff BJ, Paulus W. The effect of lorazepam on the motor cortical excitability in man. Exp brain Res 1996;109:127–35.

6. Kimiskidis VK, Papagiannopoulos S, Kazis DA, Sotirakoglou K, Vasiliadis G, Zara F, et al. Lorazepam-induced effects on silent period and corticomotor excitability. Exp Brain Res 2006;173:603–11.

7. Di Lazzaro V, Pilato F, Dileone M, Ranieri F, Ricci V, Profice P, et al. GABAA receptor subtype specific enhancement of inhibition in human motor cortex. J Physiol 2006;575(Pt 3):721–6.

8. Di Lazzaro V, Rothwell JC. Corticospinal activity evoked and modulated by non-invasive stimulation of the intact human motor cortex. J Physiol 2014;592:4115–28.

9. Rossini PM, Burke D, Chen R, Cohen LG, Daskalakis Z, Di Iorio R, et al. Non-invasive electrical and magnetic stimulation of the brain, spinal cord, roots and peripheral nerves: Basic principles and procedures for routine clinical and research application. An updated report from an I.F.C.N. Committee. Clin Neurophysiol 2015;126:1071–107.

10. Yu FH, Catterall WA. Overview of the voltage-gated sodium channel family. Genome Biol 2003;4:207.

11. Lee HW, Seo HJ, Cohen LG, Bagic A, Theodore WH. Cortical excitability during prolonged antiepileptic drug treatment and drug withdrawal. Clin Neurophysiol 2005;116:1105–12.

12. Ziemann U, Lonnecker S, Steinhoff BJ, Paulus W. Effects of antiepileptic drugs on motor cortex excitability in humans. Ann Neurol 1996;40:367–78.

13. Hadzic M, Jack A, Wahle P. Ionotropic glutamate receptors: Which ones, when, and where in the mammalian neocortex. J Comp Neurol 2017;525:976–1033.

14. Ziemann U, Reis J, Schwenkreis P, Rosanova M, Strafella A, Badawy R, et al. TMS and drugs revisited 2014. Clin Neurophysiol 2015;126:1847–68.

15. Lazzaro V Di, Oliviero A, Profice P, Pennisi MA, Pilato F, Zito G, et al. Ketamine Increases Human Motor Cortex Excitability to Transcranial Magnetic Stimulation. J Physiol 2003;547:485–96.

16. Di Lazzaro V, Ziemann U. The contribution of transcranial magnetic stimulation in the functional evaluation of microcircuits in human motor cortex. Front Neural Circuits 2013;7:1–9.

17. Boroojerdi B, Battaglia F, Muellbacher W, Cohen L. Mechanisms influencing stimulus-response properties of the human corticospinal system. Clin Neurophysiol 2001;112:931–7.

18. Stetkarova I, Kofler M. Differential effect of baclofen on cortical and spinal inhibitory circuits. Clin Neurophysiol 2013;124:339–45.

19. Pierantozzi M, Grazia Marciani M, Giuseppina Palmieri M, Brusa L, Galati S, Donatella Caramia M, et al. Effect of Vigabatrin on motor responses to transcranial magnetic stimulation: An effective tool to investigate in vivo GABAergic cortical inhibition in humans. Brain Res 2004;1028:1–8.

20. Werhahn KJ, Kunesch E, Noachtar S, Benecke R, Classen J. Rapid Report Differential effects on motorcortical inhibition induced by blockade of GABA uptake in humans. J Physiol. 1999; 517: 591–597.

21. Kujirai T, Caramia MD, Rothwell JC, Day BL, Thompson PD, Ferbert A, et al. Corticocortical inhibition in human motor cortex. J Physiol 1993;471:501–19.

22. Di Lazzaro V, Restuccia D, Oliviero A, Profice P, Ferrara L, Insola A, et al. Magnetic transcranial stimulation at intensities below active motor threshold activates intracortical inhibitory circuits. Exp brain Res 1998;119:265–8.

23. Ilić T V., Meintzschel F, Cleff U, Ruge D, Kessler KR, Ziemann U. Short-interval paired-pulse inhibition and facilitation of human motor cortex: The dimension of stimulus intensity. J Physiol 2002;545:153–67.

24. Müller-Dahlhaus JFM, Liu Y, Ziemann U. Inhibitory circuits and the nature of their interactions in the human motor cortex - a pharmacological TMS study. J Physiol 2008;586:495–514.

25. Teo JTH, Terranova C, Swayne O, Greenwood RJ, Rothwell JC. Differing effects of intracortical circuits on plasticity. Exp Brain Res 2009;193:555–63.

26. Fisher RJ, Nakamura Y, Bestmann S, Rothwell JC, Bostock H. Two phases of intracortical inhibition revealed by transcranial magnetic threshold tracking. Exp Brain Res 2002;143:240–8.

27. Stagg CJ, Bestmann S, Constantinescu AO, Moreno Moreno L, Allman C, Mekle R, et al. Relationship between physiological measures of excitability and levels of glutamate and GABA in the human motor cortex. J Physiol 2011;589:5845–55.

28. Sanger TD, Garg RR, Chen R. Interactions between two different inhibitory systems in the human motor cortex. J Physiol 2001;530:307–17.

29. Peurala SH, M. Müller-Dahlhaus JF, Arai N, Ziemann U. Interference of short-interval intracortical inhibition (SICI) and short-interval intracortical facilitation (SICF). Clin Neurophysiol 2008;119:2291–7.

30. Paulus W, Classen J, Cohen LG, Large CH, Di Lazzaro V, Nitsche M, et al. State of the art: Pharmacologic effects on cortical excitability measures tested by transcranial magnetic stimulation. Brain Stimul. 2008;1:151–63.

31. Mohammadi B, Krampfl K, Petri S, Bogdanova D, Kossev A, Bufler J, et al. Selective and nonselective benzodiazepine agonists have different effects on motor cortex excitability. Muscle and Nerve. 2006;33:778–84.

32. Schwenkreis P, Witscher K, Janssen F, Addo A, Dertwinkel R, Zenz M, et al. Influence of the N-methyl-D-aspartate antagonist memantine on human motor cortex excitability. Neurosci Lett. 1999;270:137–40.

33. Ziemann U, Chen R, Cohen LG, Hallett M. Dextromethorphan decreases the excitability of the human motor cortex. Neurology 1998;51:1320–4.

34. Nakamura H, Kitagawa H, Kawaguchi Y, Tsuji H. Intracortical facilitation and inhibition after transcranial magnetic stimulation in conscious humans. J Physiol 1997;498:817–23.

35. Valls-Solé J, Pascual-Leone A, Wassermann EM, Hallett M. Human motor evoked responses to paired transcranial magnetic stimuli. Electroencephalogr Clin Neurophysiol Evoked Potentials. 1992;85:355–64.

36. McDonnell MN, Orekhov Y, Ziemann U. The role of GABAB receptors in intracortical inhibition in the human motor cortex. Exp Brain Res 2006;173:86–93.

37. Ziemann U, Tergau F, Wassermann EM, Wischer S, Hildebrandt J, Paulus W. Demonstration of facilitatory I wave interaction in the human motor cortex by paired transcranial magnetic stimulation. J Physiol 1998;511:181–90.

38. Di Lazzaro V, Rothwell JC, Oliviero a, Profice P, Insola a, Mazzone P, et al. Intracortical origin of the short latency facilitation produced by pairs of threshold magnetic stimuli applied to human motor cortex. Exp Brain Res 1999;129:494–9.

39. Ziemann U, Tergau F, Wischer S, Hildebrandt J, Paulus W. Pharmacological control of facilitatory I-wave interaction in the human motor cortex. A paired transcranial magnetic stimulation study. Electroencephalogr Clin Neurophysiol - Electromyogr Mot Control 1998;109:321–30.

40. Conde V, Tomasevic L, Akopian I, Stanek K, Saturnino GB, Thielscher A, et al. The non-transcranial TMS-evoked potential is an inherent source of ambiguity in TMS-EEG studies. Neuroimage 2019;185:300–12.

41. Biabani M, Fornito A, Mutanen TP, Morrow J, Rogasch NC. Characterizing and minimizing the contribution of sensory inputs to TMS-evoked potentials. Brain Stimul 2019;12:1537–52.

42. ter Braack EM, de Vos CC, van Putten MJAM. Masking the Auditory Evoked Potential in TMS–EEG: A Comparison of Various Methods. Brain Topogr 2015;28:520–8.

43. Belardinelli P, Biabani M, Blumberger DM, Bortoletto M, Casarotto S, David O, et al. Reproducibility in TMS–EEG studies: A call for data sharing, standard procedures and effective experimental control. Brain Stimul 2019;12:787–90.

44. Siebner HR, Conde V, Tomasevic L, Thielscher A, Bergmann TO. Distilling the essence of TMS-evoked EEG potentials (TEPs): A call for securing mechanistic specificity and experimental rigor. Brain Stimul 2019;12:1051–4.

45. Premoli I, Castellanos N, Rivolta D, Belardinelli P, Bajo R, Zipser C, et al. TMS-EEG Signatures of GABAergic Neurotransmission in the Human Cortex. J Neurosci 2014;34:5603–12.

46. Nikouline V, Ruohonen J, Ilmoniemi RJ. The role of the coil click in TMS assessed with simultaneous EEG. Clin Neurophysiol 1999;110:1325–8.

47. Premoli I, Rivolta D, Espenhahn S, Castellanos N, Belardinelli P, Ziemann U, et al. Characterization of GABAB-receptor mediated neurotransmission in the human cortex by paired-pulse TMS-EEG. Neuroimage 2014;103:152–62.

48. Mäki H, Ilmoniemi RJ. The relationship between peripheral and early cortical activation induced by transcranial magnetic stimulation. Neurosci Lett 2010;478:24–8.

49. Casula EP, Rocchi L, Hannah R, Rothwell JC. Effects of pulse width, waveform and current direction in the cortex: A combined cTMS-EEG study. Brain Stimul 2018;11:1063–70.

50. Darmani G, Bergmann TO, Zipser C, Baur D, Müller‐Dahlhaus F, Ziemann U. Effects of antiepileptic drugs on cortical excitability in humans: A TMS-EMG and TMS-EEG study. Hum Brain Mapp 2019;40:1276-1289.

51. Premoli I, Biondi A, Carlesso S, Rivolta D, Richardson MP. Lamotrigine and levetiracetam exert a similar modulation of TMS-evoked EEG potentials. Epilepsia 2017;58:42–50.

52. Lang N, Rothkegel H, Peckolt H, Deuschl G. Effects of lacosamide and carbamazepine on human motor cortex excitability: A double-blind, placebo-controlled transcranial magnetic stimulation study. Seizure. 2013;22(9):726–30.

53. Chen R, Samii A, Caños M, Wassermann EM, Hallett M. Effects of phenytoin on cortical excitability in humans. Neurology 1997;49:881–3.

54. Sommer M, Gileles E, Knappmeyer K, Rothkegel H, Polania R, Paulus W. Carbamazepine reduces short-interval interhemispheric inhibition in healthy humans. Clin Neurophysiol 2012;123:351–7.

55. Ossemann M, de Fays K, Bihin B, Vandermeeren Y. Effect of a single dose of retigabine in cortical excitability parameters: A cross-over, double-blind placebo-controlled TMS study. Epilepsy Res 2016;126:78–82.

56. Inghilleri M, Berardelli A, Marchetti P, Manfredi M. Effects of diazepam, baclofen and thiopental on the silent period evoked by transcranial magnetic stimulation in humans. Exp Brain Res 1996;109:467–72.

57. Lang N, Sueske E, Hasan A, Paulus W, Tergau F. Pregabalin exerts oppositional effects on different inhibitory circuits in human motor cortex: A double-blind, placebo-controlled transcranial magnetic stimulation study. Epilepsia. 2006;47:813–9.

58. Sohn YH, Kaelin-Lang A, Jung HY, Hallett M. Effect of levetiracetam on human corticospinal excitability. Neurology 2001;57:858–63.

59. Solinas C, Lee YC, Reutens DC. Effect of levetiracetam on cortical excitability: A transcranial magnetic stimulation study. Eur J Neurol 2008;15:501–5.

60. Reis J, Wentrup A, Hamer HM, Mueller HH, Knake S, Tergau F, et al. Levetiracetam influences human motor cortex excitability mainly by modulation of ion channel function - A TMS study. Epilepsy Res 2004;62:41–51.

61. Wischer S, Paulus W, Sommer M, Tergau F. Piracetam affects facilitatory I-wave interaction in the human motor cortex. Clin Neurophysiol 2001;112:275–9.

62. Reis J, Tergau F, Hamer HM, Müller HH, Knake S, Fritsch B, et al. Topiramate selectively decreases intracortical excitability in human motor cortex. Epilepsia. 2002;43:1149–56.

**Supplementary Tables**

**Supplementary Table 1. Effects of common AEDs on TMS parameters in healthy participants.** The grouping of AEDs is crude as most are considered to have multiple mechanisms of actions, some of which remain unknown.

| **AED** | **rMT** | **CSP** | **SICI** | **ICF** | **LICI** | **SICF** | **TEP** |
| --- | --- | --- | --- | --- | --- | --- | --- |
| **Na^+^ channel blockers (CBZ, LCM, LTG)** | ↑^11,12,17,39,50–53^ | ↑ (CBZ)^12^ or ND (PHT)^53^ | ↓ (LTG and CBZ)^12,50^ or ND (CBZ, LCM, PHT)^12,17,52–54^ | ND^12,17,52–54^ | ND^53^ | ND^39^ | ↓P25, ↓P180 (CBZ)^50^; ↑N45, ↓P180 (LTG)^51^ |
| **Kv7 channel agonist (RTG)** | ↑^55^ | NT | ND^55^ | ND^55^ | ND^55^ | ND^55^ | NT |
| **Benzodiazepines (DZP, LZP)** | ND^5–7,17,39^, ↑ (DZP) ^24^ | ↑ intensity rMT^6^ and 120% of rMT^5^; ↓ 125% of rMT^56^ and 200% rMT^6^ | ↑ (DZP,LZP)^7,23–25^ or ND (LZP)^17,54^ | ↓ (LZP, DZP)^5,31,54^ or ND (LZP)^17^ | ND^24,25,31^ | ↓ ^23,39^ | ↑N45, ↓N100 (DZP)^45^ |
| **AEDs increasing GABA synaptic availability (TGB, VGB)** | ND^12,19,20,39,50^ | ↑ ^19,20^ | ↓ (TGB)^20^ or ND (VGB, TGB)^12,19,50^ | ↑ (TGB)^20^ or ↓(VGB)^12^ | ↑ (TGB)^20^; ND (VGB)^19^ | ↓^39^ | ND^50^ |
| **HVA Ca^2+^ channel blockers GBP, PGB)** | ND ^12,39,57^ | ↑ ^12^ | ↑ (GBP) ^50^; or ↓ (PGB) ^57^ | ↓ (GBP ^12^ or ND (PGB) ^57^ | ↑^57^ | ND^39,50^ | NT |
| **SV2A ligands (BRV, LEV)** | ND^50,58^ or ↑^51,59,60^ | ND ^58,60^ or ↑ ^59^ | ND ^50,58–60^ | ND^58–60^ | NT | NT | ↑N45, ↓P180^51^; ↓N100^50^ |
| **PRC** | NT | NT | NT | NT | NT | ↓ amplitude and latency of peaks; ^61^ | NT |
| **TPM (multiple effects)** | ND^62^ | ND^62^ | ↑ ^62^ | ND^62^ | NT | NT | NT |

Abbreviations: BRV – brivaracetam; CBZ – carbamazepine; DZP – diazepam; GBP – gabapentin; HVA – high-voltage activated; LCM – lacosamide; LTG – lamotrigine; LEV – levetiracetam; LZP – lorazepam; PRC – piracetam; PBG – pregabalin; RTG – retigabine; SV2A – synaptic vesicle glycoprotein 2A; TGB – tiagabine; TPM – topiramate; VGB – vigabatrin; ND – no difference; NT – not tested
